# Intestinal perforation secondary to abdominal angiostrongyliasis in an older infant with suspected extraintestinal involvement

**DOI:** 10.1093/omcr/omag088

**Published:** 2026-06-08

**Authors:** Hugo-Malik Cherif, Nadia Suazo, Gustavo Fontecha, Flor Girón

**Affiliations:** Facultad de Ciencias Médicas, Universidad Nacional Autónoma de Honduras, Tegucigalpa, Honduras; Departamento de Patología, Hospital Escuela/Facultad de Ciencias Médicas, Universidad Nacional Autónoma de Honduras, Tegucigalpa, Honduras; Instituto de Investigaciones en Microbiología, Facultad de Ciencias, Universidad Nacional Autónoma de Honduras, Tegucigalpa, Honduras; Departamento de Patología, Facultad de Ciencias Médicas, Universidad Nacional Autónoma de Honduras, Tegucigalpa, Honduras

**Keywords:** *Angiostrongylus spp*, Angiostrongyliasis, Honduras, parasitology, tropical medicine

## Abstract

Angiostrongyliasis is a parasitic disease caused by *Angiostrongylus costaricensi*s (Latin America) and *A. cantonensis* (Asia/Pacific). Abdominal angiostrongyliasis often mimics appendicitis, challenging clinical diagnosis and requiring histopathological confirmation. We report a severely malnourished infant in Honduras presenting with 10 days of severe abdominal pain and vomiting, complicated by respiratory insufficiency and seizures. Brain CT revealed cortical atrophy, suggesting a chronic underlying neurologic condition. Laboratory tests showed leukocytosis, lymphocytosis, and massive eosinophilia. Exploratory laparotomy for suspected appendicitis identified an ileal perforation, requiring intestinal resection. Histopathology confirmed non-caseating granulomas containing *A. costaricensis* larvae within the mesenteric arteriovenous plexus, alongside scattered eggs. This is the first confirmed human case of abdominal angiostrongyliasis with suspected, unconfirmed extraintestinal involvement in the Olancho Department of Honduras. These findings emphasize the critical need for increased clinical awareness and public health measures against this overlooked parasitic infection.

**Statement of Significance**

This case documents the first confirmed *Angiostrongylus costaricensis* infection in Olancho, Honduras, presenting as intestinal perforation in an infant. For pathologists and trainees, this report underscores the diagnostic complexity of abdominal angiostrongyliasis, which frequently mimics appendicitis or Crohn’s disease. It illustrates the essential morphological features for diagnosis: intense eosinophilic vasculitis, granulomatous inflammation, and the specific identification of intra-arterial larvae and eggs. Additionally, the findings suggest rare disseminated disease in the context of severe malnutrition. This report highlights the critical need to maintain a high index of suspicion for parasitic etiologies in eosinophil-rich granulomatous lesions of the gastrointestinal tract, ensuring accurate diagnosis in regions where this neglected tropical disease is underrecognized.

## Introduction

Angiostrongyliasis is a parasitic disease caused by nematodes of the genus *Angiostrongylus*, primarily *A. costaricensis* in Latin America and *A. cantonensis* in Asia and the Pacific, with sporadic reports of the latter in the Americas [1, 2]. *A. costaricensis* causes abdominal angiostrongyliasis, a zoonosis. in which larvae penetrate the intestinal wall and migrate to mesenteric arteries, inducingeosinophilic vasculitis and granulomatous inflammation.

Clinically, the disease often mimics acute appendicitis or inflammatory bowel disease, presenting with abdominal pain, fever, and gastrointestinal symptoms [3]. Because the parasite does not complete its reproductive cycle in humans, parasitological diagnosis is not feasible; therefore, confirmation relies on histopathological identification of larvae or eggs within granulomatous lesions [4]. This limitation, combined with low clinical suspicion, contributes to underdiagnosis.

Although reported in Central America [3], the true burden remains underestimated. Severe complications such as intestinal ischemia, perforation, or extraintestinal involvement (pulmonary or neurological) are rare [4–6], especially in pediatric cases.

We report a severe case of abdominal angiostrongyliasis in an older infant with intestinal perforation and suggestive of extraintestinal involvement. To our knowledge, this is the first confirmed case from Olancho, Honduras.

## Case report

A 1-year-old infant from a rural area in Olancho, Honduras presented to the emergency department with a 10-day history of abdominal pain accompanied by vomiting and progressive abdominal distension. Upon admission, his vital signs were profoundly unstable: heart rate 186 bpm, respiratory rate 12 bpm, temperature 36.2°C, blood pressure 70/50 mmHg, and oxygen saturation 86% on room air. He was unresponsive to external stimuli, exhibiting active tonic–clonic convulsions. Physical examination revealed severe emaciation, lethargy, cyanosis, tachycardia, weak peripheral pulses and delayed capillary refill (>3 s). The respirations were shallow and irregular. The abdomen was markedly distended and tympanic, with absent bowel sounds and diffuse abdominal rigidity consistent with peritonitis. He also had hyponatremia and left-sided hemiparesis.

Initial laboratory tests revealed marked leukocytosis driven by lymphocytosis and massive eosinophilia, along with evidence of severe malnutrition (Table 1).

**Table 1:** Laboratory results at admission in the emergency department.

**Exam**	**Result**	**Reference range**
**WBC**	16.01 × 10^3^/μL	4.00–10.00 × 10^3^/μL
**Neutrophils**	3.61 × 10^3^/μL	2.00–7.00 × 10^3^/μL
**Lymphocytes**	8.05 × 10^3^/μL	0.80–4.00 × 10^3^/μL
**Monocytes**	0.62 × 10^3^/μL	0.12–1.20 × 10^3^/μL
**Eosinophils**	3.52 × 10^3^/μL	0.02–0.50 × 10^3^/μL
**Basophils**	0.03 × 10^3^/μL	0.00–0.10 × 10^3^/μL
**Stool guaiac**	Positive	—
**ALP**	265 U/L	46–116 U/L
**TGO**	31 U/L	15–37 U/L
**TGP**	76 U/L	12–78 U/L
**Creatinine**	0.06 mg/dL	0.5–1.3 mg/dL
**Albumin**	2.5 g/dL	3.4–5.1 g/dL
**Sodium**	122 mmol/L	136–145 mmol/L
**Potassium**	4 mmol/L	3.4–5.1 mmol/L
**Phosphorus**	6.2 mmol/L	2.5–4.9 mmol/L

**Figure 1:**
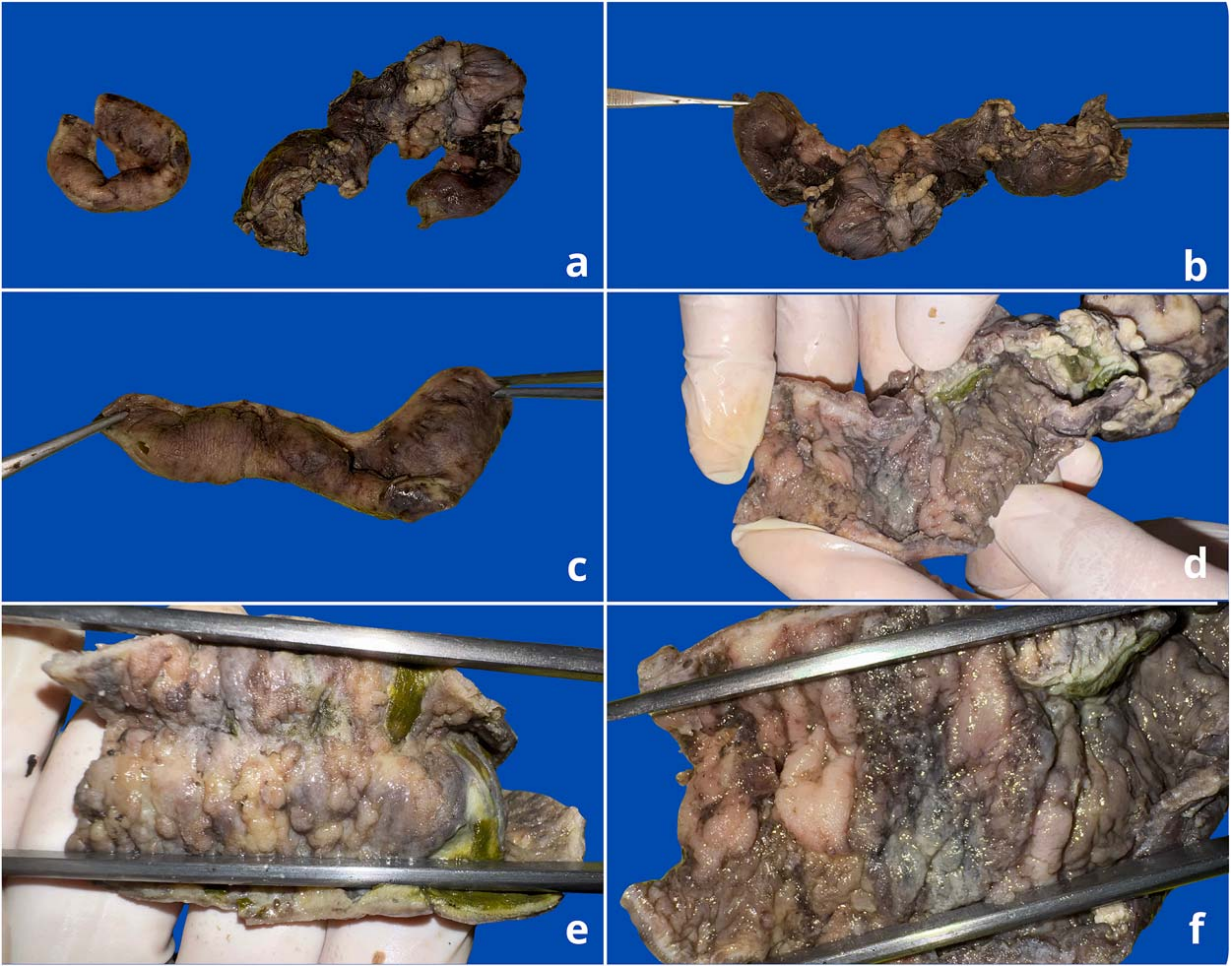
Macroscopic features of the resected ileum. (a–c) Entire segment of resected ileum showing marked thickening of the intestinal wall, loss of pliability, and areas of serosal opacity. (d) Granulomatous and necrotic areas adjacent to the site of perforation, with irregular nodularity of the mucosal and submucosal surfaces. (e) Multiple coalescing granulomatous nodules distributed along the intestinal wall. (f) Extensive transmural necrotic areas with dark, ischemic discoloration and loss of normal mucosal architecture.

Brain CT demonstrated left hemispheric atrophy, likely from a previous structural defect. Because acute appendicitis was suspected, the patient underwent exploratory laparotomy, which revealed ileal perforation. An intestinal resection with terminal ileostomy was performed on day 1 of hospitalization. Gross examination of the resected specimen showed granulomatous areas near the perforation site, reminiscent of Crohn’s-like lesions, as well as zones of intestinal necrosis (Figure 1).

**Figure 2:**
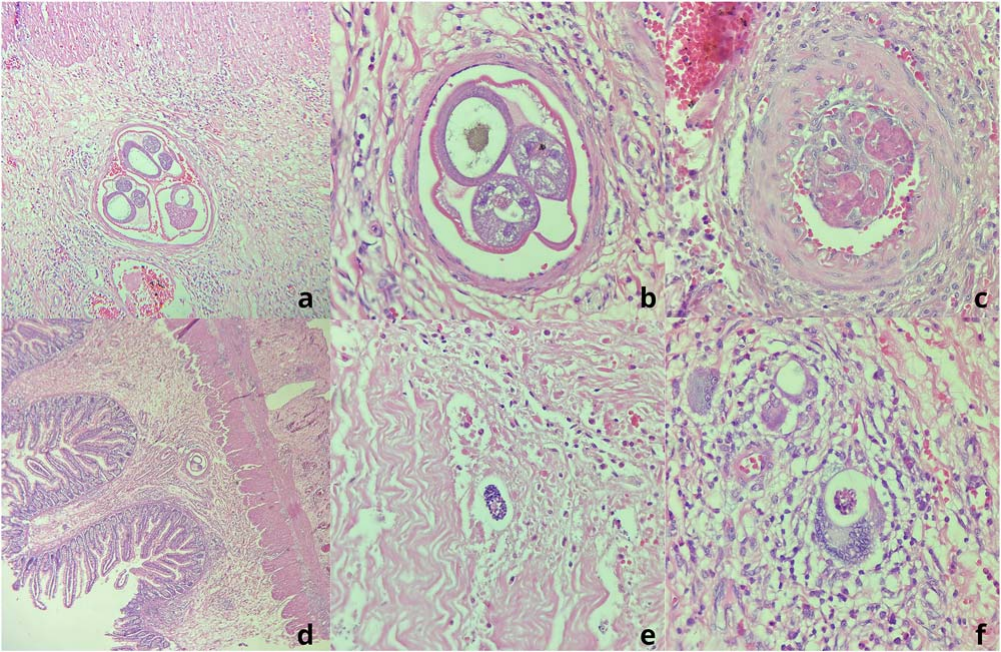
Histopathological findings of the resected ileum (H&E staining). (a–c) Multiple *Angiostrongylus costaricensis* fifth-stage (L5) larvae located within mesenteric and submucosal blood vessels, producing marked eosinophilic vasculitis. (d) Transmural eosinophilic and granulomatous inflammation with larval structures adjacent to the muscularis propria. (e) Numerous *Angiostrongylus* spp. eggs distributed in the submucosa and interstitial stroma. (f) Multinucleated giant cell exhibiting active phagocytosis of parasitic eggs, surrounded by dense eosinophilic infiltration.

H&E-stained sections demonstrated an ulcerated mucosa and intense transmural eosinophilic infiltration. Within the blood vessels of the submucosa and *muscularis propria*, multiple fifth-stage (L5) larvae of *A. costaricensis* were identified, together with numerous eggs consistent with *Angiostrongylus* spp. Granulomatous chronic inflammation was present throughout the intestinal wall, including Langhans-type and foreign-body–type multinucleated giant cells, some of which showed phagocytosed parasitic eggs. Eosinophils and neutrophils were also abundant in the serosa. Extensive transmural hemorrhagic–ischemic necrosis was observed. (Figure 2).

The patient’s only identified possible exposure was consumption of untreated water and undercooked rice atole, with no other risk factors for angiostrongyliasis; rodent exposure, inadequate household sanitation and similar illness in the family and community were all excluded.

The acute clinical management focused on supportive care and symptom control. Medical treatment included gastric protection with esomeprazole, symptomatic control for pain/fever (paracetamol and dipyrone), seizures/spasms (diazepam and menalgil), and nutritional support.

Anthelmintic therapy was withheld to prevent exacerbating the local inflammatory response that would result from parasite death within the mesenteric arteriovenous plexus. The patient required 5 weeks of hospitalization, including 10 days of critical care.

Despite the severity, the outcome was favorable, with complete recovery at 6 months post-hospitalization.

## Discussion

Angiostrongyliasis is a parasitic infection caused by the nematode *Angiostrongylus costaricensis*. Rodents serve as definitive hosts, while mollusks act as intermediate hosts in which the parasite develops from first-stage (L1) to third-stage (L3) larvae. Humans acquire the infection accidentally through ingestion of L3 larvae present in contaminated vegetables, mollusk mucus, or rodent feces. After intestinal penetration, larvae migrate to mesenteric arteries, where they mature to L5 stage and deposit eggs. As humans are dead-end hosts, the life cycle cannot be completed [6].

*A. costaricensis* typically causes abdominal infection, but ectopic migration of eggs or larvae can occur, leading to serious complications like granulomatous meningoencephalitis or eosinophilic pneumonitis. This parasite is endemic in Latin America, including Honduras, where its presence in various hosts has been well-documented [1, 2, 3]. In humans, the clinical presentation includes abdominal pain, fever and eosinophilia, and may mimic acute appendicitis, ileitis, or inflammatory bowel disease [4]. Histopathologically, the key lesion is intense eosinophilic granulomatous inflammation and eosinophilic vasculitis of the intestinal wall and mesenteric vessels. This reaction, driven by eggs in the stroma and larvae in blood vessels, can cause stenosis, ischemia, or perforation [6, 7].

While *A. costaricensis* typically causes abdominal disease and *A. cantonensis* causes neuroangiostrongyliasis, ectopic *A. costaricensis* infections challenge this distinction. Experimental evidence shows that *A. costaricensis* can cause pulmonary lesions and cerebral invasion, suggesting hematogenous or lymphatic spread. Though rare, these respiratory or neurological symptoms must be considered in patients like ours who have compatible epidemiological exposure alongside abdominal symptoms [8].

In a profoundly malnourished infant presenting with eosinophilia, intestinal perforation, and neurological decline, the differential diagnosis is broad. Infectious etiologies such as severe abdominal tuberculosis, complicated amebiasis, or *Strongyloides stercoralis* hyperinfection must be considered. Inflammatory bowel conditions mimicking acute appendicitis, as well as profound metabolic derangements secondary to severe protein-calorie malnutrition, also form critical components of the differential.

Since no globally available serological tests exist, the definitive diagnosis of *A. costaricensis* relies on histopathological identification of larvae and eggs in the tissue. Molecular assays have been developed but are not yet accessible in many endemic settings, including Honduras [9]. Therefore, diagnosis depends on clinical suspicion, epidemiological exposure, eosinophilia, and imaging findings.

Treatment is mainly supportive, with surgery required for complications such as perforation. Anthelmintic therapy is controversial, as parasite death may intensify inflammatory responses. Corticosteroids have been used for severe inflammation in ectopic disease, but formal guidelines are lacking [10].

This case highlights the need for strengthened regional epidemiological surveillance, updated public health policies, and molecular diagnostic capacity in reference laboratories.

Preventive strategies are essential for reducing the burden of this neglected parasitic disease. The severe clinical presentation, combined with profound malnutrition, may have facilitated systemic involvement but could not be proved in our resource-limited setting.
